# Mulberroside A Alleviates Scopolamine-Induced Cognitive Deficits by Suppressing Neuroinflammation and Oxidative Stress via the *Dubosiella*-Associated Microbiota–Gut–Brain Axis

**DOI:** 10.3390/biology15131030

**Published:** 2026-06-28

**Authors:** Jin Li, Shirui Cheng, Wenqi Zhang, Shourong Qiao, Luzhi Zhang, Mengxu Yao, Yunxia Zhang, Biao Wang, Changjing Wu

**Affiliations:** 1 College of Life Sciences and Agronomy, Zhoukou Normal University, Zhoukou 466001, China; 15938983243@163.com (S.C.); 18547751030@163.com (W.Z.); 13137420683@163.com (S.Q.); 19711306337@163.com (L.Z.); 15233618369@163.com (M.Y.); yunxiaflight@163.com (Y.Z.); 20231018@zknu.edu.cn (B.W.); 2 Fuxi Laboratory, Zhoukou Normal University, Zhoukou 466001, China; 3 Key Laboratory of Agricultural Animal Genetics, Breeding and Reproduction of Ministry of Education, College of Animal Science and Technology & College of Veterinary Medicine, Huazhong Agricultural University, Wuhan 430070, China; 4 Field Observation and Research Station of Green Agriculture in Dancheng County, Zhoukou 466001, China

**Keywords:** mulberroside A, Alzheimer’s disease, microbiota–gut–brain axis, *Dubosiella*, neuroinflammation, oxidative stress

## Abstract

Mulberroside A (MsA) possesses neuroprotective effects, but whether it alleviates Alzheimer’s disease (AD)-like cognitive impairment through the microbiota–gut–brain axis remains unclear. Using a scopolamine-induced mouse model of acute cognitive impairment (male ICR mice, *n* = 10/group), we demonstrated that daily administration of MsA (10, 20, and 30 mg/kg/day) for 5 weeks significantly ameliorated cognitive performance in novel object recognition and Morris water maze tests. At the optimal dose (30 mg/kg/day), MsA suppressed hippocampal microglial activation, reduced pro-inflammatory cytokines (IL-6, IL-1β, TNF-α), and attenuated oxidative stress by decreasing malondialdehyde (MDA) while restoring superoxide dismutase (SOD) and glutathione (GSH) levels. MsA also strengthened intestinal barrier integrity (ZO-1, occludin) and significantly altered the gut microbiota, notably increasing the beneficial genus *Dubosiella*. Brain metabolomics indicated that MsA reversed scopolamine-induced metabolic disturbances, mainly restoring phospholipid balance. Correlation analysis demonstrated a strong gut–brain connection, with *Dubosiella* abundance positively associated with neuroprotective phospholipids and negatively with stress markers. Furthermore, fecal microbiota transplantation from MsA-treated donors successfully replicated these behavioral improvements in recipient mice, underscoring the functional involvement of the reshaped microbiome rather than a simple autonomous recovery. These results suggest that MsA alleviates AD-like cognitive impairment by reducing neuroinflammation and oxidative stress through microbiota remodeling, enhancing the intestinal barrier, and modulating the *Dubosiella*-associated gut–metabolite–brain axis, making MsA a promising multi-target nutraceutical for ameliorating AD-like cognitive deficits.

## Simple Summary

Alzheimer’s disease is a progressive neurodegenerative disorder that causes memory loss, cognitive decline, and great burden on patients and families. Although many studies have focused on the brain, increasing evidence suggests that changes in the gut may also contribute to disease development. In this study, we examined whether mulberroside A, a natural compound derived from mulberry, could protect against scopolamine-induced Alzheimer’s disease-like cognitive impairment in mice. Our results showed that mulberroside A improved learning and memory, reduced inflammatory damage and oxidative stress in the brain, and protected the intestinal barrier. We also found that mulberroside A changed the composition of gut microbes, especially increasing beneficial bacteria such as *Dubosiella*, and helped restore abnormal metabolism in the brain. Importantly, fecal microbiota transplantation from treated mice reproduced the protective effects, indicating that gut microbiota changes play a key role in the action of mulberroside A. These findings suggest that mulberroside A may improve brain health by acting through the microbiota-gut–brain axis and could be a promising natural compound for the prevention and amelioration of Alzheimer’s disease-like cognitive decline.

## 1. Introduction

Alzheimer’s disease (AD) is a devastating neurodegenerative disorder characterized by progressive cognitive decline, memory loss, and behavioral disturbances, representing the most common cause of dementia worldwide [1,2]. Pathologically, AD is defined by the accumulation of extracellular amyloid-beta (Aβ) plaques and intracellular neurofibrillary tangles composed of hyperphosphorylated tau protein, alongside chronic neuroinflammation, oxidative stress, and neuronal loss [3]. Despite extensive research, current therapeutic strategies primarily offer symptomatic relief, and no effective disease-modifying treatments are yet available [4,5]. This urgent unmet medical need has driven research towards exploring novel etiological factors and therapeutic targets beyond the classical amyloid and tau hypotheses, leading to increased attention on the intricate interplay between peripheral systems and brain health, particularly the microbiota-gut–brain axis (MGBA) [6,7]. Emerging evidence suggests that dysbiosis of the gut microbiota, metabolic disorders, and compromised intestinal barrier integrity may significantly contribute to the initiation and progression of AD-like cognitive decline by promoting systemic inflammation and oxidative stress that ultimately impact central nervous system (CNS) function [8,9,10].

The MGBA represents a complex bidirectional communication system involving neural, endocrine, immune, and metabolic pathways, which plays a crucial role in maintaining brain homeostasis and modulating cognitive functions [11]. Dysregulation of the MGBA, often characterized by altered gut microbial composition, impaired gut barrier function (leading to a “leaky gut”), and subsequent systemic inflammation, has been consistently observed in AD patients and animal models [9,12]. These peripheral disturbances are widely hypothesized to facilitate the translocation of bacterial products (e.g., lipopolysaccharides, LPS) and pro-inflammatory cytokines from the gut into the bloodstream and subsequently across a compromised blood–brain barrier (BBB), potentially activating resident glial cells and exacerbating neuroinflammation within the brain [13,14]. Moreover, gut microbiota dysbiosis can impact brain metabolism through altered production of microbial metabolites such as short-chain fatty acids (SCFAs) and tryptophan derivatives, which can influence neurogenesis, neurotransmitter synthesis, and mitochondrial function, all critical for cognitive health [15,16]. Consequently, targeting the MGBA to restore gut eubiosis, enhance intestinal barrier integrity, and mitigate peripheral inflammation presents a promising multifaceted strategy for managing cognitive deficits [17].

Mulberroside A (MsA), a natural polyphenolic glycoside isolated from *Morus alba* L., has garnered considerable interest due to its diverse pharmacological properties, including antioxidant, anti-inflammatory, and neuroprotective effects [18,19,20,21]. In our previous study, MsA was shown to exert comprehensive neuroprotection by alleviating scopolamine-induced cognitive deficits, cholinergic dysfunction, Aβ accumulation, and tau hyperphosphorylation [22]. These findings established MsA as a promising multi-target candidate for ameliorating AD-like deficits; however, they primarily focused on central pathological events and did not address whether its neuroprotective effects may also involve peripheral regulatory mechanisms. Notably, MsA exhibits relatively low oral bioavailability and limited direct blood–brain barrier permeability [23], suggesting that its in vivo efficacy may not rely solely on direct brain exposure but could also be mediated through modulation of peripheral homeostasis, particularly the MGBA. Crucially, MsA has been shown to co-regulate peripheral mucosal and central nervous pathways. Peripherally, MsA remodels gut microbiota, promotes SCFA synthesis, and fortifies the intestinal barrier, thereby preventing systemic endotoxemia from propagating neuroinflammation [24]. Centrally, MsA alleviates hippocampal senescence by downregulating p16, p21, and Rb [21], while suppressing NF-κB/MAPK pathways to inhibit microglial activation and pro-inflammatory cytokine release [25]. Based on this rationale, and considering the emerging importance of gut dysbiosis, intestinal barrier dysfunction, and microglial activation in neurodegenerative conditions, it is reasonable to hypothesize that MsA may confer additional benefits by remodeling the gut microbiota, preserving intestinal barrier integrity, and reprogramming brain metabolism.

Although transgenic models are ideal for capturing chronic progressive pathology, the scopolamine (SCOP)-induced model serves as a well-established and widely accepted pharmacological paradigm for evaluating cognitive-enhancing interventions. By blocking muscarinic acetylcholine receptors, SCOP reliably precipitates acute cholinergic dysfunction, neuroinflammation, and oxidative stress, thereby mimicking key early-stage behavioral and biochemical features of AD-like cognitive decline. Recent studies have successfully utilized this model to decipher gut microbiota dysbiosis and peripheral–central metabolic crosstalk along the gut–brain axis [26,27,28], demonstrating its high sensitivity and appropriateness for investigating MGBA-targeted nutraceutical interventions without the confounding genetic variations in transgenic systems. Therefore, the present study was designed to investigate the systemic effects of MsA against SCOP-induced acute cognitive impairment in a mouse model, with a particular focus on gut microbiota restoration, intestinal barrier fortification, and their potential regulatory connection with the attenuation of neuroinflammation and oxidative stress via the gut–microbiota–metabolite–brain axis.

## 2. Materials and Methods

### 2.1. Materials and Reagents

Scopolamine (SCOP, CAS: 6533-68-2) and mulberroside A (MsA, purity > 98%, CAS: 102841-42-9) were purchased from Aladdin Biochemical Technology Co., Ltd. (Shanghai, China). Donepezil (DNP, CAS: 120011-70-3), used as a positive control, was obtained from TargetMol (Shanghai, China). All assay kits and chemicals, with their catalog numbers and manufacturers, are listed in Table S1 in Section S2.1 of the Supplementary Materials.

### 2.2. Scopolamine-Induced Mice and Treatment

**Figure 1:**
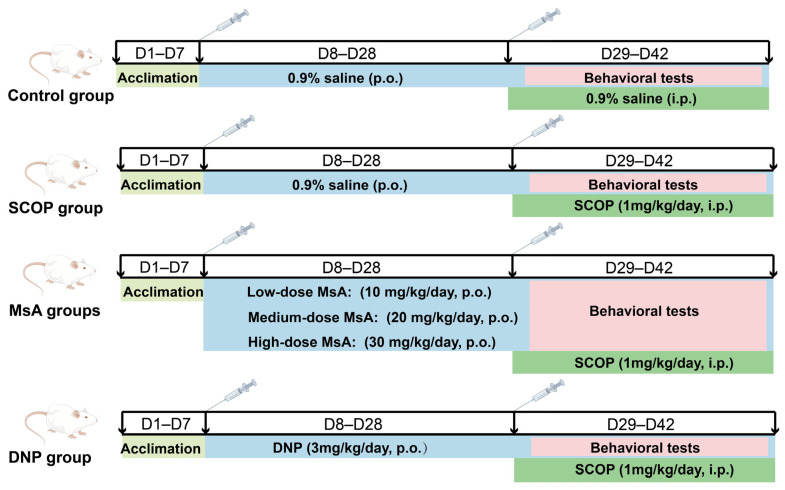
Schematic diagram of the mice experimental design and treatment schedule.

Male ICR mice (4–6 weeks old) were procured from the Model Animal Research Centre of Nanjing University (Nanjing, China). All animals were group-housed (4 per cage) under standard conditions with a 12 h light/dark cycle, ambient temperature of 23 ± 2 °C, and ad libitum access to food and water. After one week of acclimatization, the mice were randomly allocated into six experimental groups (*n* = 10 per group) using a computer-generated random number sequence to ensure unbiased allocation: control (0.9% saline), scopolamine (SCOP, 1 mg/kg/day, i.p.), SCOP + mulberroside A (MsA, 10, 20, and 30 mg/kg/day, p.o.), and SCOP + donepezil (DNP, 3 mg/kg/day, p.o.). The entire experimental procedure lasted 42 days, comprising three distinct phases: (1) acclimation (D1–D7): all mice were habituated to the environment; (2) pre-treatment (D8–D28): mice in the MsA and DNP groups received daily oral administration for 21 days to ensure steady-state concentrations; (3) behavioral testing and induction (D29–D42): mice continued to receive their respective treatments via gavage, and scopolamine was administered intraperitoneally 30 min before behavioral sessions for 14 consecutive days. The first-line clinical acetylcholinesterase inhibitor, donepezil, was utilized as a positive control to facilitate the evaluation of the restorative effects of MsA on cholinergic function and cognitive performance, as well as to verify the reliability of the experimental system. To guarantee experimental rigor and reproducibility, all behavioral evaluations, tissue harvesting, and subsequent biochemical assays were conducted in a strictly blinded manner, with investigators remaining completely unaware of the specific treatment group assignments. The detailed experimental timeline is illustrated in Figure 1. The experimental protocol was approved by the Animal Welfare Committee of Zhoukou Normal University (Approval No: ZKNU-20240132; Date: 3 January 2024), and all procedures were conducted in accordance with the ethical guidelines for the care and use of laboratory animals.

### 2.3. New Object Recognition (NOR) Test

The NOR test was conducted to evaluate the recognition memory of the mice, comprising an acquisition (training) phase and a testing phase. The experimental apparatus consisted of a gray open-field arena (30 cm × 30 cm × 30 cm). The objects utilized included two identical cubes (familiar objects, F) and a cylinder of distinct shape and color (novel object, N). (1) Acquisition phase: Each mouse was placed into the arena containing two identical familiar objects (F) and allowed to explore freely for 10 min. (2) Testing phase: After a 24 h retention interval, one of the familiar objects was replaced with the novel object (N). The mouse was reintroduced into the arena, and the time spent exploring both the novel and familiar objects was recorded over a 10 min period. Exploratory behavior was defined as directing the nose toward the object at a distance of ≤2 cm, sniffing, or physically contacting it. Following each trial, the arena and objects were thoroughly decontaminated with 75% ethanol to eliminate potential olfactory cues. To quantify exploratory preference, novel object performance was calculated as the exploration time ratio spent on the novel object: Novel object performance = N/(N + F). Recognition memory was assessed using the discrimination index (DI), calculated as follows: DI = (N − F)/(N + F), where N and F represent the time spent exploring the novel and familiar objects, respectively. Animal trajectories and locomotor activities were recorded and processed using the ANY-maze video tracking system (version 7.49, Stoelting Co., Wood Dale, IL, USA).

### 2.4. Novel Location Recognition (NLR) Test

The NLR test was utilized to assess spatial memory and location recognition capabilities. This task was performed in the same gray open-field arena (30 cm × 30 cm × 30 cm) utilizing two completely identical objects (cubes). (1) Acquisition phase: Two identical objects (A and B) were symmetrically placed in adjacent corners of the arena. Mice were allowed 10 min of free exploration to habituate to the objects and their spatial configuration. (2) Testing phase: Following a 24 h interval, object A was displaced to a novel location while object B remained in its original position. The mice were reintroduced to the arena, and the time spent exploring the objects in both the novel and familiar locations was recorded for 10 min. All animal trajectories and exploration times were recorded and analyzed using the ANY-maze video tracking system (version 7.49, Stoelting Co., Wood Dale, IL, USA). The criteria for exploratory behavior and the decontamination protocols (using 75% ethanol) were identical to those described for the NOR test. To evaluate spatial preference, novel location performance was calculated as the exploration time ratio spent on the displaced object: Novel location performance = N/(N + F). Spatial memory was also evaluated using the location discrimination index (DI), calculated as: DI = (N − F)/(N + F), where N and F denote the time spent exploring the displaced (novel location) and non-displaced (familiar location) objects, respectively. This index serves as a reliable indicator of the ability of the mice to recognize and retain changes in spatial locations.

### 2.5. Morris Water Maze (MWM) Test

Spatial cognitive abilities of the mice were assessed via the MWM task. The apparatus comprised a circular water pool conceptually partitioned into four equal quadrants, with a submerged escape platform consistently positioned in a fixed target quadrant. On the initial day (habituation phase), each mouse was released into the water facing the pool wall of the third quadrant and given 60 s for free exploration. Subjects failing to discover the platform within this period were manually guided to it and allowed to remain there for 10 s to acquire spatial cues. During the subsequent 5-day acquisition phase, the mice were subjected to four daily trials, starting from pseudo-randomly assigned quadrants. The time required to navigate to the hidden platform (escape latency) was monitored. If an animal failed to reach the target within 60 s, it was physically directed to the platform, and its latency was capped at 60 s. To evaluate memory consolidation, a probe trial was executed on day 6. The platform was extracted from the pool, and the mice were introduced from the quadrant diametrically opposite the previous platform location. During the 60 s probe test, the time elapsed in the target quadrant, the frequency of platform-site crossings, and the swimming distance within the target zone were captured. All behavioral metrics and swim trajectories were digitally recorded and analyzed utilizing the ANY-maze video tracking system (version 7.49, Stoelting Co., Wood Dale, IL, USA).

### 2.6. Tissue and Fecal Sample Collection

Upon the completion of all behavioral assessments, fresh fecal pellets were collected from the mice, immediately transferred into sterile cryovials, and stored at −80 °C for subsequent gut microbiota analysis. Subsequently, the mice were subjected to an overnight fast with free access to water. On the following day, mice in the control group received an equivalent volume of sterile saline (G4702, Servicebio Technology Co., Ltd., Wuhan, China), whereas mice in the model and treatment groups received an intraperitoneal (i.p.) injection of scopolamine (1 mg/kg). After a 30 min interval to replicate the acute pharmacological window and central muscarinic blockade experienced by the animals during the behavioral trials, the animals were euthanized via cervical dislocation, followed by immediate dissection for tissue harvesting. The whole brains were rapidly excised on ice, from which the cerebral cortex and hippocampus were carefully isolated. Concurrently, segments of the colon, jejunum, and ileum were harvested. Portions of the brain and intestinal tissues were fixed in 4% paraformaldehyde for subsequent histomorphological evaluation. The remaining tissue specimens were snap-frozen in liquid nitrogen and stored at −80 °C for further molecular and biochemical assays.

### 2.7. H&E Staining

Following euthanasia, full-thickness segments of the ileum and jejunum were immediately excised, gently flushed with ice-cold phosphate-buffered saline (PBS) (G0002, Servicebio Technology Co., Ltd., Wuhan, China) to remove intestinal contents, and fixed in 4% paraformaldehyde overnight at room temperature. The fixed tissues were subsequently dehydrated through a graded ethanol series, cleared in xylene, and embedded in paraffin wax. Serial sections (4–5 μm thick) were cut using a microtome, mounted onto glass slides, and dried. For routine hematoxylin and eosin (H&E) staining, the sections were deparaffinized in xylene and rehydrated through descending grades of ethanol to distilled water. The slides were then stained with hematoxylin for 3–5 min, differentiated in 1% HCl-ethanol solution (prepared in-house) for several seconds, rinsed in running tap water for bluing, and counterstained with eosin for 1–3 min. Finally, the stained sections were dehydrated, cleared in xylene, and permanently mounted with a neutral synthetic resin. The pathomorphological alterations of the ileal and jejunal tissues were observed, photographed, and analyzed using an optical microscope (Olympus, Tokyo, Japan).

### 2.8. Immunohistochemical Analysis

For immunohistochemical analysis, whole brains were rapidly excised on ice after euthanasia and fixed in 4% paraformaldehyde (G1101, Servicebio Technology Co., Ltd., Wuhan, China). Brain tissues containing the hippocampal region were then paraffin-embedded and sectioned for subsequent Iba1 staining. Tissue sections underwent deparaffinization and rehydration, followed by the quenching of endogenous peroxidase activity using 0.3% H_2_O_2_ (prepared from 30% H_2_O_2_, CAS: 7722-84-1, Aladdin Biochemical Technology Co., Ltd., Shanghai, China) in methanol for 15 min. Antigen retrieval was achieved by heating the slides in citrate buffer (pH 6.0) using a microwave oven, and then blocking non-specific binding with 5% normal goat serum (G1208, Servicebio Technology Co., Ltd., Wuhan, China) for 1 h at 37 °C. The sections were incubated overnight at 4 °C with a primary anti-Iba1 antibody (GB114490, Servicebio Technology Co., Ltd., Wuhan, China). Following PBS washing, the sections underwent incubation with a biotinylated goat anti-rabbit secondary antibody at 37 °C for 30 min. Visualization of the antigen–antibody complex was achieved using the avidin-biotin-peroxidase complex (ABC) reagent (AB_2336382, Vector Laboratories, Newark, CA, USA) and a 3,3′-diaminobenzidine (DAB) chromogen kit (G1212, Servicebio Technology Co., Ltd., Wuhan, China). Finally, the slides were counterstained with hematoxylin, dehydrated, cleared, and mounted with neutral resin. Iba1-positive microglia in the hippocampus were observed and captured using an optical microscope (Olympus, Tokyo, Japan). For the quantification of Iba1-positive microglia, three coronal sections containing the dorsal hippocampus were evaluated per animal (*n* = 3 mice/group). Using ImageJ software (version 1.54f, National Institutes of Health, Bethesda, MD, USA). The definitive boundaries of CA1, CA3, and DG were manually delineated. Morphometric quantification was performed across each subregion to eliminate sampling bias. To ensure objectivity, both image acquisition and subsequent digital analysis were conducted in a strictly blinded manner by an independent investigator.

### 2.9. Biochemical Assays

Cryopreserved cortical tissue samples were mechanically homogenized in ice-cold physiological saline (0.9% NaCl) (G4702, Servicebio Technology Co., Ltd., Wuhan, China). Following centrifugation at 4 °C, the resulting supernatants were carefully collected for subsequent biochemical evaluations. To assess the extent of oxidative stress, the malondialdehyde (MDA) content, superoxide dismutase (SOD) activity, and reduced glutathione (GSH) levels in the tissues were systematically quantified using specific commercial kits purchased from Nanjing Jiancheng Bioengineering Institute (Nanjing, China; MDA: Cat. No. A003-1; SOD: Cat. No. A001-3; GSH: Cat. No. A006-2-1), in strict adherence to the manufacturer’s instructions. All experimental procedures were conducted in strict adherence with the corresponding assay kit instructions. The OD of each well was measured at assay-specific wavelengths using a fully automated microplate reader (Thermo Fisher Scientific, Waltham, MA, USA). Finally, the specific concentrations or enzymatic activities of these biochemical parameters were calculated according to the standard curves generated for each respective assay.

### 2.10. Quantitative Real-Time PCR

After euthanasia, the whole brain was rapidly removed on ice, and the hippocampus was carefully dissected from both hemispheres. The isolated hippocampal tissues were snap-frozen in liquid nitrogen and stored at −80 °C until RNA extraction. Total RNA was isolated from the mouse hippocampal tissues utilizing the TRIzol reagent (R0016, Beyotime, Shanghai, China) in accordance with standard recommended protocols. Subsequent to the extraction process, the yield and purity of the obtained RNA were quantified via a NanoDrop 2000 spectrophotometer (Thermo Fisher Scientific, Waltham, MA, USA), while its structural integrity was thoroughly verified through agarose gel electrophoresis. First-strand cDNA synthesis was then executed employing a commercial reverse transcription kit (G3337, Servicebio Technology Co., Ltd., Wuhan, China). For the quantitative assessment of target gene expression, qRT-PCR was carried out using a SYBR Green PCR Master Mix on a LightCycler platform (Roche, Basel, Switzerland). GAPDH was adopted as the endogenous internal control to normalize the amplification data. The relative mRNA transcription levels of the inflammatory markers, specifically *IL-1β*, *IL-6*, and *TNF-α*, in tissue samples were calculated applying the standard 2^−ΔΔCt^ method. The specific primer sequences utilized for these amplifications are detailed in Table 1.

### 2.11. Western Blotting Analysis

Total proteins from mouse colon tissues were extracted using RIPA lysis buffer (G2002, Servicebio Technology Co., Ltd., Wuhan, China) containing protease and phosphatase inhibitors (PMSF, P0100; Phosphatase Inhibitor Cocktail, P1260; Beijing Solarbio Science & Technology Co., Ltd., Beijing, China). Segments of the colon were homogenized to assess the expression of intestinal barrier-related proteins (ZO-1 and Occludin). Following concentration determination via a BCA protein assay kit (Cat. No. P0010; Beyotime Biotechnology, Shanghai, China), the protein samples were denatured at 95 °C for 5 min. Equivalent amounts of protein were separated by SDS-PAGE and subsequently electro-transferred onto nitrocellulose (NC) membranes. The membranes were blocked with 5% non-fat milk and then incubated overnight at 4 °C with primary antibodies against ZO-1 (1:1000; Servicebio Technology Co., Ltd., Wuhan, China; Cat. No. GB15195) and Occludin (1:1000; Thermo Fisher Scientific, Waltham, MA, USA; Cat. No. 33-1500). The membranes were incubated with corresponding HRP-conjugated secondary antibodies for 1 h at room temperature. Finally, protein bands were visualized using ECL reagents and captured by a chemiluminescence imaging system (Bio-Rad, Hercules, CA, USA). Densitometric analysis was performed using ImageJ software, with GAPDH (Servicebio, Wuhan, China; Cat. No. GB15002; 1:5000) and β-Tubulin (Servicebio, Wuhan, China; Cat. No. GB122667; 1:5000) used as loading controls.

### 2.12. 16S rRNA Gene Sequencing Analysis

Total genomic DNA was extracted from cryopreserved mouse fecal samples using a commercial DNA extraction kit (D4015, Omega Bio-tek, Norcross, GA, USA), and its overall yield and integrity were validated. The V3-V4 hypervariable regions of the bacterial 16S rRNA gene were subsequently amplified via PCR, purified, and utilized to construct sequencing libraries. Following equimolar pooling, paired-end sequencing was performed on an Illumina MiSeq platform (Illumina, San Diego, CA, USA). Raw sequencing data were processed using the QIIME2 bioinformatics pipeline, where high-quality sequences were denoised via DADA2 to generate Amplicon Sequence Variants (ASVs) and taxonomically annotated against the SILVA reference database. Ultimately, comprehensive downstream evaluations—including α-diversity indices, β-diversity (visualized by PCoA and NMDS), LEfSe differential analysis, and phenotypic correlation assessments—were systematically conducted to elucidate the specific structural alterations in the gut microbiota.

### 2.13. Untargeted Metabolomics Analysis

For untargeted metabolomic profiling, metabolites from the cryopreserved brain tissues (hippocampus and cerebral cortex) were extracted via homogenization and sonication in an ice-cold solvent mixture. Following centrifugation at 4 °C, the supernatants were vacuum-concentrated, reconstituted in a methanol-water system, and filtered through a 0.22 μm membrane. Pooled quality control (QC) samples were simultaneously prepared to monitor system stability. Chromatographic separation was performed on a Vanquish UHPLC system (Thermo Fisher Scientific, Waltham, MA, USA) equipped with an ACQUITY UPLC BEH Amide column (Waters Corporation, Milford, MA, USA), utilizing a gradient elution of aqueous ammonium acetate/ammonia (mobile phase A) and acetonitrile (mobile phase B) at a flow rate of 0.5 mL/min. Mass spectrometry acquisition was executed on a Q Exactive HFX high-resolution mass spectrometer equipped with an electrospray ionization (ESI) source (Thermo Fisher Scientific, Waltham, MA, USA), operating in both positive and negative ion modes. Data-dependent acquisition (DDA) was employed with resolutions set to 60,000 for full MS scans and 7500 for MS/MS spectra. Finally, the raw mass spectra were systematically processed utilizing XCMS software (version 3.16.1) for peak picking, alignment, and retention time correction, ultimately yielding a robust data matrix of metabolic features (comprising retention times, *m*/*z* values, and peak intensities) for subsequent multidimensional statistical analyses.

### 2.14. Fecal Microbiota Transplantation (FMT)

To explore the potential involvement of the gut microbiota in the neuroprotective effects of MsA, a separate FMT experiment was conducted using a new batch of naive mice as recipients. The donor feces used for transplantation were collected from the Control and MsA-treated mice described previously in Section 2.2. Specifically, donor mice received daily oral administration of MsA for 35 consecutive days, including a 21-day pre-treatment phase and a 14-day behavioral evaluation phase (as outlined in Figure 1). Fresh donor fecal pellets were collected on Day 42, approximately 24 h after the final dose of MsA and following the completion of behavioral tests, to capture a chronically adapted microbial profile rather than transient, acute drug effects. Following acclimation, ICR mice were randomly allocated into Control, SCOP, MsA-FMT, and DNP groups. To ensure successful exogenous bacterial engraftment, mice in the MsA-FMT group received a 7-day broad-spectrum antibiotic pretreatment to effectively deplete their endogenous gut microbiota. Under strict sterile conditions, fresh feces (80–100 mg) collected from MsA-treated donor mice were homogenized in 600 μL of ice-cold sterile PBS, thoroughly vortexed for 3–5 min, and centrifuged at 3000 rpm for 3 min. The resulting microbiota-enriched supernatant was orally administered (100 μL per mouse) to the MsA-FMT recipients daily from the cessation of antibiotic treatment until the experimental endpoint. Simultaneously, mice in the other groups received an equal volume of sterile PBS to control for gavage-induced stress. Concurrently, scopolamine (1 mg/kg, i.p.) was utilized to induce the cognitive deficit model, and donepezil (3 mg/kg, p.o.) served as the positive control in the DNP group. 

### 2.15. Statistics

Statistical analyses were performed using GraphPad Prism version 10 (GraphPad Software, La Jolla, CA, USA). Data are shown as the mean ± standard error of the mean (SEM), with each dot on the bars denoting a unique sample measurement. Groups were compared using one-way or two-way ANOVA, with Dunnett’s post hoc test for multiple comparisons as needed. Statistical differences were set as follows: * *p* < 0.05, ** *p* < 0.01, *** *p* < 0.001.

## 3. Results

### 3.1. MsA Ameliorates Cognitive Deficits in SCOP-Treated Mice

**Figure 2:**
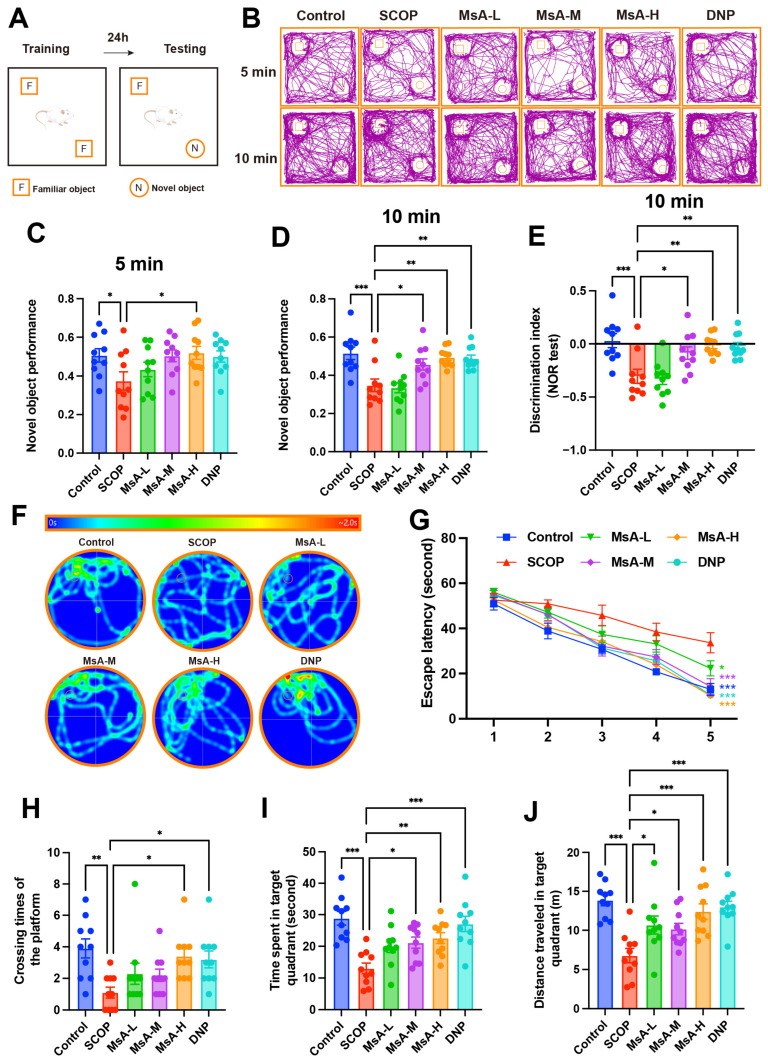
MsA mitigates SCOP-treated deficits in learning and memory. (**A**) Schematic diagram of the NOR test. (**B**–**E**) Behavioral evaluations in the NOR test, including representative movement tracking images of mice exploring familiar and novel objects ((**B**), where the irregular purple lines represent the movement trajectories of the mice, the outer orange squares indicate the testing arenas, and the inner orange dashed circles define the object exploration zones), quantitative analysis of the novel object performance at 5 min (**C**) and 10 min (**D**), and the discrimination index at 10 min (**E**). (**F**–**J**) Spatial memory assessments in the MWM test, comprising representative swimming heatmaps from the probe trial ((**F**), where the small circles within the arenas indicate the location of the hidden target platform), escape latency across the 5-day training trials (**G**), crossing times of the platform (**H**), time spent in the target quadrant (**I**), and distance traveled in the target quadrant (**J**). Data are expressed as mean ± SEM, *n* = 10. * *p* < 0.05, ** *p* < 0.01, *** *p* < 0.001 compared with the SCOP group.

To evaluate the restorative effects of MsA on recognition memory, the NOR test was performed according to the experimental paradigm (Figure 2A). As illustrated by the representative movement tracks (Figure 2B), SCOP exposure severely impaired the exploratory preference of mice for the novel object. Quantitative analysis revealed that SCOP-treated mice exhibited a significant reduction in novel object performance at both the 5 min (Figure 2C) and 10 min (Figure 2D) observation windows compared to the Control group. However, these memory deficits were effectively reversed by MsA treatment. Specifically, while MsA-H showed significant restorative effects as early as the 5 min interval (Figure 2C), both MsA-M and MsA-H, as well as DNP, significantly improved novel object performance at the 10 min window (Figure 2D). Consistently, the discrimination index at 10 min (Figure 2E) was profoundly diminished in the SCOP group but was significantly rescued following MsA-M, MsA-H, and DNP interventions, indicating a robust recovery of recognition memory.

Spatial learning and memory retention were subsequently assessed utilizing the MWM task. During the 5-day acquisition training, SCOP-challenged mice displayed notably prolonged escape latencies, whereas therapeutic interventions with MsA and DNP significantly accelerated their spatial learning curves (Figure 2G). In the probe trial, representative swimming heatmaps (Figure 2F) visually confirmed that MsA-treated mice searched more intensively within the target area. Further quantitative assessments demonstrated that SCOP administration severely reduced the number of platform crossings (Figure 2H), the time spent in the target quadrant (Figure 2I), and the distance traveled within the target quadrant (Figure 2J). Conversely, MsA administration comprehensively mitigated these spatial memory impairments. Notably, while the distance traveled within the target quadrant was significantly increased across all MsA dosage groups (MsA-L, MsA-M, and MsA-H) (Figure 2J), significant improvements in platform crossing times (Figure 2H) and target quadrant duration (Figure 2I) were primarily observed in the MsA-M and MsA-H groups. Collectively, these behavioral findings strongly suggest that MsA possesses significant neuroprotective potential against SCOP-induced cognitive dysfunction.

Considering the overall behavioral performance, MsA-H was selected for subsequent mechanistic investigations due to its stable and pronounced effects, and this high-dose MsA intervention is hereafter uniformly referred to as MsA in the following results and figures.

### 3.2. MsA Suppresses Hippocampal Microglial Activation in SCOP-Treated Mice

**Figure 3:**
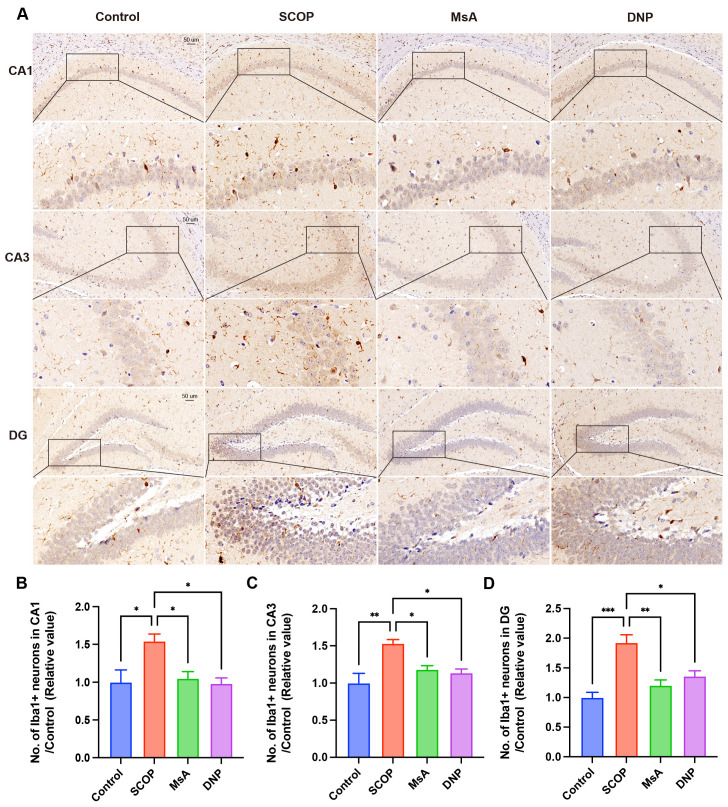
MsA inhibits hippocampal Iba1+ activation in SCOP-treated mice. (**A**) Representative immunohistochemical images of Iba1+ cells in hippocampal CA1, CA3, and DG regions, scale bar = 50 μm. (**B**–**D**) Quantitative analysis of Iba1+ cell numbers in CA1 (**B**), CA3 (**C**), and DG (**D**) regions. Data are normalized to the control group and expressed as mean ± SEM *n* = 3. * *p* < 0.05, ** *p* < 0.01, *** *p* < 0.001 compared with the SCOP group.

Microglial activation is a key event in neuroinflammatory responses. To evaluate the regulatory effect of MsA on hippocampal microglia in SCOP-treated mice, Iba1 immunohistochemical staining was performed in the CA1, CA3, and DG regions. As shown in Figure 3A, only a few Iba1-positive cells with resting-like morphology were observed in the Control group, whereas the SCOP-treated group exhibited a marked increase in Iba1-positive cells, accompanied by typical activated features, including enlarged somata and shortened processes. In contrast, MsA intervention reduced Iba1-positive cell accumulation and partially restored microglial morphology in the hippocampus. Quantitative analysis showed that Iba1-positive cell numbers were significantly elevated in the CA1, CA3, and DG regions of SCOP-treated mice compared with Control mice, while MsA treatment significantly decreased Iba1-positive cell numbers in these regions (Figure 3B–D). DNP also exerted a similar inhibitory effect on Iba1-positive microglial activation. These results indicate that MsA suppresses hippocampal microglial activation in SCOP-treated mice.

### 3.3. MsA Suppresses Inflammatory Responses and Oxidative Stress in SCOP-Treated Mice

**Figure 4:**
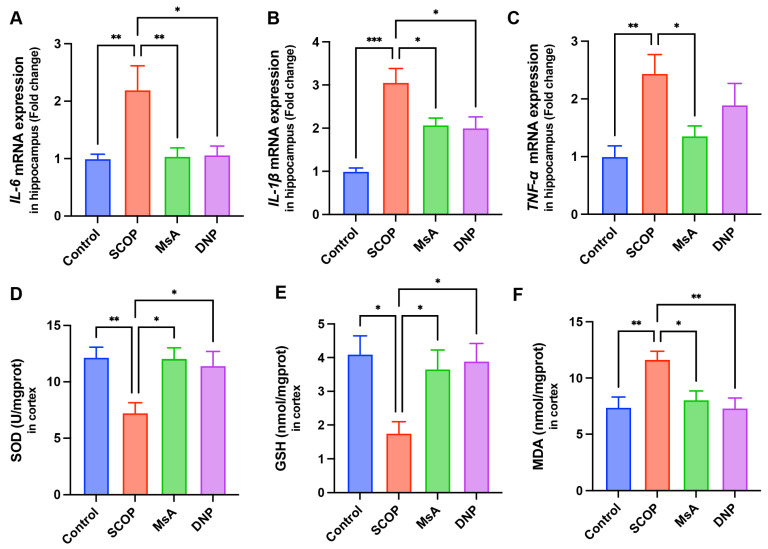
MsA attenuates neuroinflammation and oxidative stress in the brain of SCOP-treated mice. (**A**–**C**) qRT-PCR analysis of inflammatory cytokine mRNA expression in the hippocampus, including IL-6 (**A**), IL-1β (**B**), and TNF-α (**C**). (**D**–**F**) Biochemical analysis of oxidative stress-related indicators in the cortex, including SOD activity (**D**), GSH content (**E**), and MDA level (**F**). Data are expressed as mean ± SEM *n* = 5. * *p* < 0.05, ** *p* < 0.01, *** *p* < 0.001 compared with the SCOP-treated group.

To evaluate the neuroprotective effects of MsA, we examined the levels of pro-inflammatory cytokines and oxidative stress markers in the mouse brain. As shown in Figure 4A–C, the mRNA expressions of IL-6, IL-1β, and TNF-α in the hippocampus were significantly elevated following SCOP administration compared with the Control group. Notably, MsA treatment effectively downregulated the transcription of these pro-inflammatory genes, and the positive control DNP showed a similar inhibitory trend. Furthermore, we assessed the impact of MsA on redox homeostasis in the cortex (Figure 4D–F). SCOP treatment resulted in a marked decrease in the activities of antioxidant enzymes, specifically SOD and GSH, accompanied by a significant increase in the levels of MDA, a marker of lipid peroxidation. In contrast, MsA intervention significantly restored SOD activity and GSH content while reducing MDA accumulation. These results suggest that MsA possesses potent anti-inflammatory and antioxidant properties, which contribute to the mitigation of SCOP-induced neurological damage.

### 3.4. MsA Reinforces Intestinal Barrier Integrity and Restores Tight Junction Protein Expression in SCOP-Treated Mice

**Figure 5:**
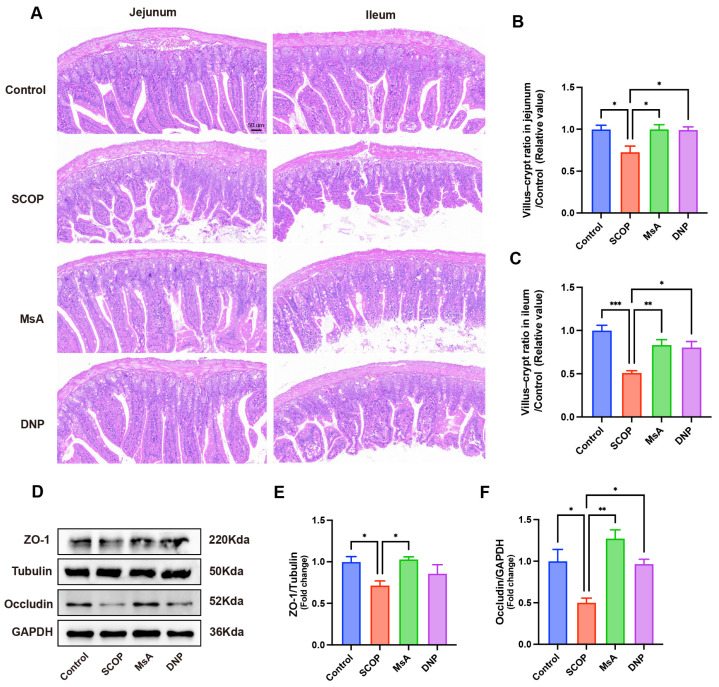
MsA alleviates intestinal barrier damage and restores tight junction protein expression in SCOP-treated mice. (**A**–**C**) Histopathological assessment of the small intestine, showing representative H&E staining images of the jejunum and ileum ((**A**), scale bar = 50 μm), and the corresponding quantification of the villus-to-crypt (V/C) ratios in the jejunum (**B**) and ileum (**C**). (**D**–**F**) Molecular analysis of colonic barrier components, featuring representative immunoblot bands of ZO-1 and Occludin (Supplementary Material Sections S1.1 and S1.2) (**D**) alongside their relative protein quantification in the colon (**E**,**F**). Data are normalized to the control group and expressed as mean ± SEM, *n* = 3. * *p* < 0.05, ** *p* < 0.01, *** *p* < 0.001 compared with the SCOP-treated group.

To assess if MsA preserves intestinal barrier integrity in SCOP-treated mice, we examined small intestinal histopathology and colonic tight junction protein levels. H&E staining revealed that the jejunum and ileum of Control mice maintained relatively intact mucosal architecture with well-organized villi. In contrast, SCOP-treated mice displayed evident intestinal mucosal damage, characterized by shortened villi and disrupted villus morphology. Quantitative analysis further showed that SCOP significantly decreased the villus-to-crypt ratios in both the jejunum and ileum, whereas MsA treatment markedly restored these ratios, indicating an improvement in small intestinal structural integrity. DNP treatment showed a comparable protective tendency (Figure 5A–C). Consistently, Western blot analysis revealed that SCOP exposure reduced the expression of the tight junction proteins ZO-1 and Occludin in colon tissues. MsA administration significantly restored the levels of both ZO-1 and Occludin, suggesting enhanced epithelial barrier function. DNP also partially improved tight junction protein expression (Figure 5D–F). Collectively, these findings indicate that MsA alleviates SCOP-induced intestinal barrier impairment by preserving small intestinal morphology and restoring colonic tight junction protein expression.

### 3.5. MsA Reshapes the Gut Microbiota Landscape and Restores Microbial Homeostasis in SCOP-Treated Mice

**Figure 6:**
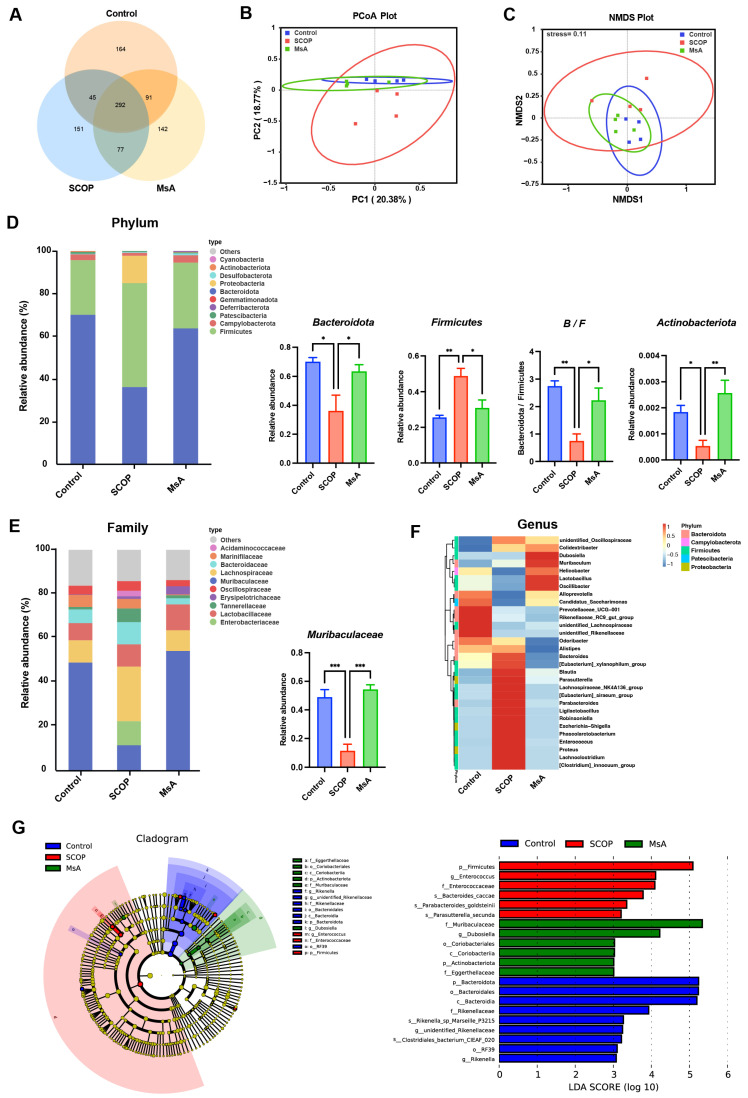
MsA ameliorates gut microbiota dysbiosis and reshapes the microbial community in SCOP-treated mice. (**A**) Venn diagram illustrating the distribution of shared and unique OTUs among the groups. (**B**,**C**) Beta diversity assessment via PCoA (**B**) and NMDS (**C**) based on Bray–Curtis distances. (**D**) Phylum-level composition, featuring stacked bar plots and statistical analysis of Bacteroidota, Firmicutes, the B/F ratio, and Actinobacteriota. (**E**) Family-level distribution and quantitative analysis of Muribaculaceae abundance. (**F**) Clustering heatmap of the top 30 differential genera, showcasing the relative abundance shifts across groups. (**G**) LEfSe analysis highlighting the taxonomic biomarkers, including a cladogram and LDA scores (LDA > 3.0). Data are presented as mean ± SEM, *n* = 4. * *p* < 0.05, ** *p* < 0.01, *** *p* < 0.001 compared with the SCOP-treated group.

To evaluate the impact of MsA on microbial ecology, 16S rRNA sequencing was conducted on fecal samples. Venn diagram analysis revealed substantial differences in the distribution of unique OTUs across groups, with 292 core OTUs shared among all samples (Figure 6A). Beta diversity analysis, performed via PCoA and NMDS, demonstrated a distinct spatial clustering of the microbial communities; specifically, SCOP-treated mice exhibited a clear separation from the Control group, whereas MsA administration significantly shifted the microbial profile back toward the Control cluster, indicating a robust restoration of the community structure (Figure 6B,C). At the taxonomic level, MsA effectively mitigated the multi-tier dysbiosis induced by SCOP. At the phylum level, SCOP-treated mice showed a notable reduction in Bacteroidota and Actinobacteriota abundance, coupled with an expansion of Firmicutes and a markedly decreased Bacteroidota/Firmicutes (B/F) ratio; these alterations were comprehensively reversed by MsA treatment (Figure 6D). Consistent with these findings, family-level analysis indicated that the abundance of Muribaculaceae, which was severely depleted in SCOP-treated mice, was robustly rescued following MsA intervention (Figure 6E). Heatmap analysis at the genus level further revealed that MsA suppressed the enrichment of opportunistic pathogens (e.g., *Enterococcus* and *Proteus*) while selectively promoting beneficial genera associated with anti-inflammatory properties, including *Dubosiella*, *Lactobacillus*, and *Alloprevotella* (Figure 6F). Finally, LEfSe analysis identified distinct microbial taxa as potential biomarkers, showing that MsA markedly remodeled the gut microbiota dysbiosis in SCOP-treated mice by enriching beneficial taxa, particularly *Dubosiella*, while SCOP treatment was associated with the enrichment of opportunistic taxa such as *Enterococcus* and *Parasutterella* (Figure 6G).

### 3.6. MsA Modulates the Brain Metabolic Landscape in SCOP-Treated Mice

**Figure 7:**
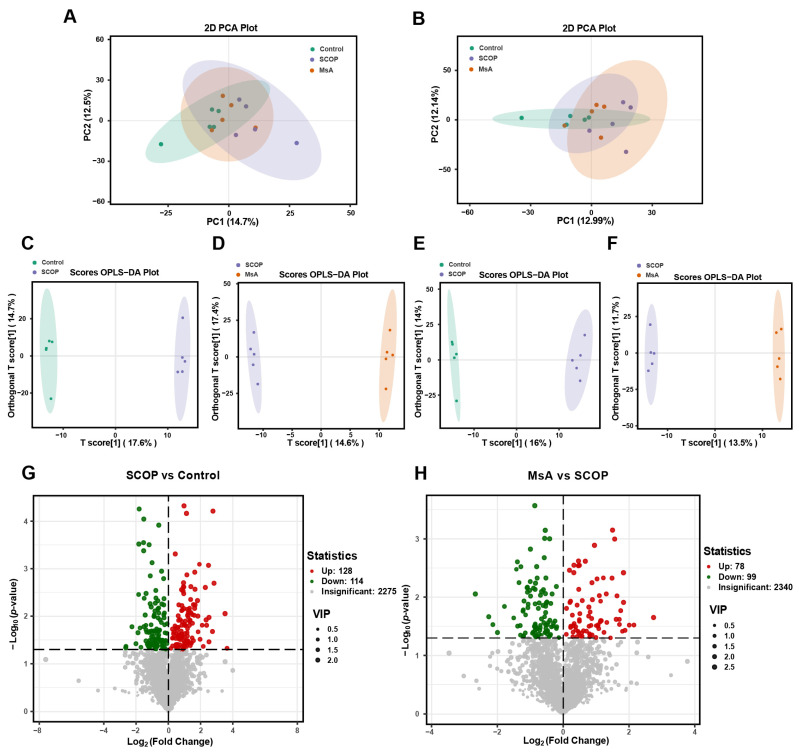
MsA modulates the brain metabolic landscape in SCOP-treated mice. (**A**,**B**) PCA score plots showcasing the distribution of metabolic profiles in positive (**A**) and negative (**B**) ion modes. (**C**–**F**) OPLS-DA score plots comparing Control vs. SCOP ((**C**): ESI^+^; (**E**): ESI^−^) and SCOP vs. MsA ((**D**): ESI^+^; (**F**): ESI^−^). (**G**,**H**) Volcano plot analysis of differential metabolites in SCOP vs. Control (**G**) and MsA vs. SCOP (**H**), with significant metabolites highlighted based on the screening criteria of VIP > 1 and *p* < 0.05. Data are derived from *n* = 5 biological replicates per group.

To further elucidate the regulatory impact of MsA on brain metabolism, non-targeted metabolomics was performed on hippocampal and cortical tissues. Unsupervised PCA demonstrated that the metabolic profiles of SCOP-treated mice were distinctly segregated from those of the Control group in both positive and negative ion modes, indicating a substantial pathological shift in the brain’s metabolic state. Notably, the metabolic trajectory of the MsA-treated group exhibited a clear shift back toward the Control cluster, suggesting that MsA effectively counteracts the metabolic deviations induced by SCOP (Figure 7A,B). To enhance class discrimination and identify stable metabolic features, supervised OPLS-DA was applied. The results showed robust separation between the Control and SCOP groups, as well as between the SCOP and MsA groups, in both ionization modes, confirming that SCOP induces a recognizable metabolic signature that is significantly remodeled by MsA intervention (Figure 7C–F). Differential metabolites were subsequently screened using the criteria of VIP > 1 and *p* < 0.05. Volcano plot analysis revealed that SCOP challenge caused widespread metabolic disturbances, involving 242 differential metabolites (128 upregulated and 114 downregulated) compared to the Control group (Figure 7G). Conversely, MsA administration significantly reprogrammed the metabolic landscape, modulating 177 metabolites (78 upregulated and 99 downregulated) relative to the SCOP-treated group (Figure 7H). Collectively, these findings suggest that MsA exerts its neuroprotective effects through comprehensive metabolic reprogramming, restoring the brain’s overall metabolic phenotype to a healthy state.

**Figure 8:**
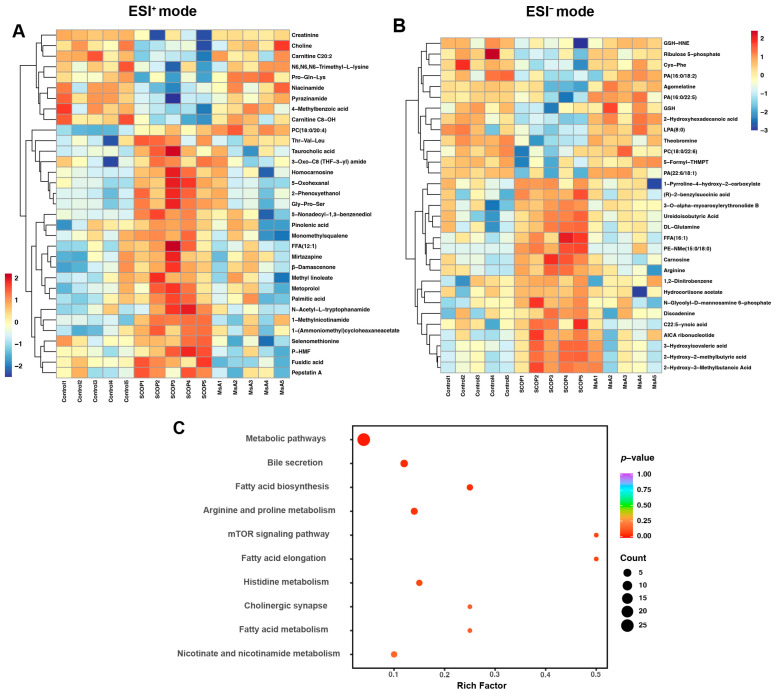
MsA targets core metabolic biomarkers and modulates metabolic pathways in SCOP-treated mice. (**A**,**B**) Hierarchical clustering heatmaps of common differential metabolites in ESI^+^ mode (*n* = 33, (**A**)) and ESI^−^ mode (*n* = 31, (**B**)). The color gradient represents Z-score normalized relative abundance, where red indicates high expression and blue indicates low expression. (**C**) KEGG pathway enrichment bubble plot of the 64 common differential metabolites. The x-axis represents the Rich Factor, bubble size corresponds to the count of enriched metabolites, and bubble color indicates the statistical significance. Data are derived from *n* = 5 biological replicates per group.

To pinpoint the specific metabolic targets of MsA, we focused on “common differential metabolites”—those significantly perturbed by SCOP and subsequently restored by MsA intervention. A total of 64 core metabolites (33 in ESI^+^ mode and 31 in ESI^−^ mode) were identified as potential biomarkers of MsA efficacy. Hierarchical clustering analysis revealed a consistent pattern across both ionization modes: samples from the MsA group clustered closely with the Control group, whereas SCOP-treated mice formed a distinct, isolated cluster characterized by systemic metabolic aberrations (Figure 8A,B). In addition, the corresponding metabolite annotations and regulatory patterns in ESI^+^ and ESI^−^ modes are summarized in Tables S2 and S3 in Sections S2.2 and S2.3 of the Supplementary Materials, respectively. To further explore the biological significance of these metabolic changes, KEGG pathway enrichment analysis was conducted on the 64 biomarkers. These metabolites were enriched in pathways including Metabolic pathways, bile secretion, fatty acid biosynthesis, arginine and proline metabolism, fatty acid elongation, the mTOR signaling pathway, histidine metabolism, the cholinergic synapse, fatty acid metabolism, and nicotinate and nicotinamide metabolism (Figure 8C). Notably, the enrichment results indicated the involvement of pathways associated with lipid metabolism, amino acid pathways, and synaptic transmission, specifically the cholinergic synapse.

### 3.7. MsA Administration Correlates with Alterations in the Gut Microbiota–Metabolite Axes in SCOP-Treated Mice

**Figure 9:**
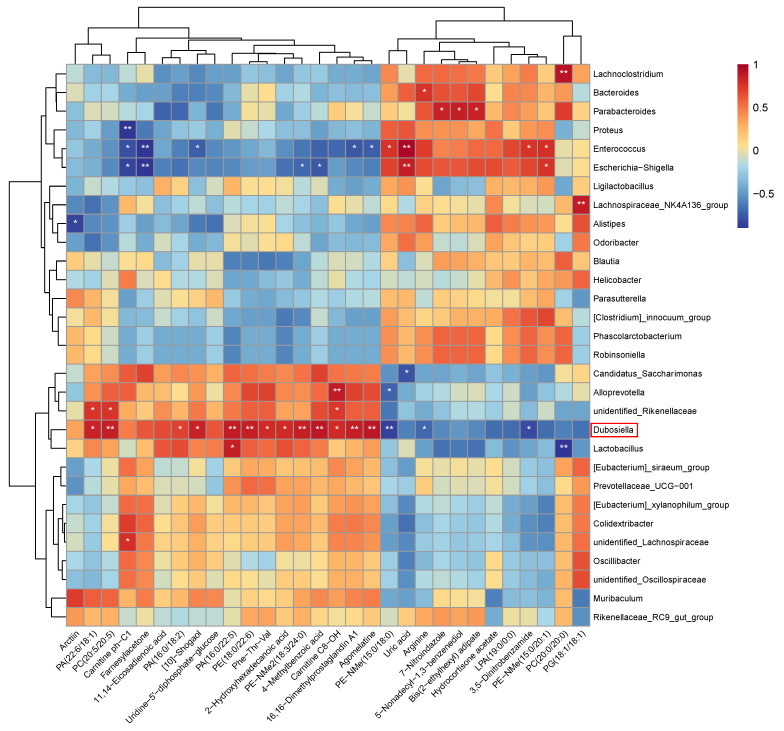
Correlation heatmap analysis between gut microbiota and differential metabolites. MsA vs. SCOP. *n* = 4, * *p* < 0.05, ** *p* < 0.01.

To explore the potential interplay between the gut microbiota and brain metabolic profiles, we performed Spearman correlation analysis between the top 30 bacterial genera and 30 core differential metabolites (Figure 9). Our analysis revealed that the beneficial genus *Dubosiella*, which was specifically enriched by MsA intervention, exhibited positive statistical associations with neuroprotective metabolites, such as PA (22:6/18:1) and PC(20:5/20:5), while maintaining a significant negative correlation with the metabolic stress marker uric acid. In stark contrast, opportunistic pathogens enriched in SCOP-treated mice, including *Enterococcus* and *Escherichia-Shigella*, displayed the opposite correlation pattern, showing negative associations with neuroprotective lipids. Additionally, *Lactobacillus* was found to be positively associated with the phospholipid component PC (20:0/20:0). Integrating these findings with the previous LEfSe results, these patterns suggest a potential associative link between MsA treatment and the gut microbiota–metabolite axis. Specifically, the enrichment of beneficial taxa like *Dubosiella* co-occurs with a trend toward the restoration of central phospholipid profiles and the suppression of oxidative stress, thereby providing preliminary, correlative evidence for the symbiotic network associated with MsA’s cognitive-enhancing properties in SCOP-treated mice.

### 3.8. MsA-FMT Improves Novel Location Recognition and Spatial Memory in SCOP-Treated Mice

**Figure 10:**
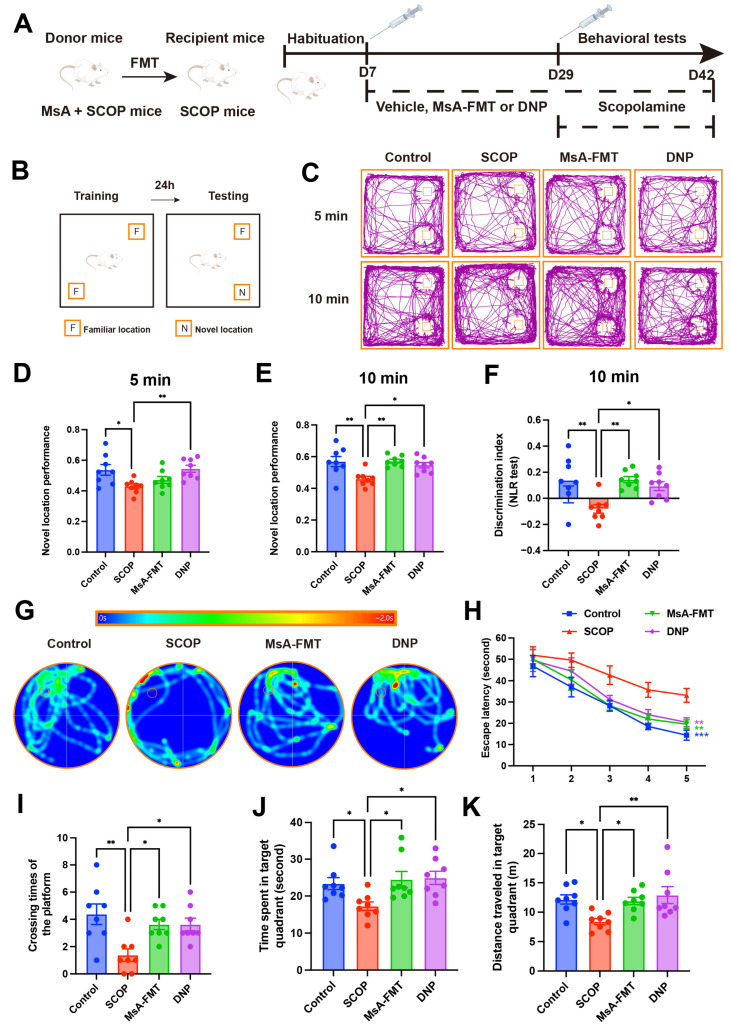
MsA-FMT improves cognitive performance in SCOP-treated mice. (**A**) Schematic illustration of the experimental timeline for FMT and subsequent behavioral assessments. (**B**,**C**) Schematic of the NLR test and representative movement trajectories at 5 and 10 min, where the irregular purple lines represent the movement trajectories of the mice, the orange squares indicate the object locations, and the orange dashed lines define the zone boundaries. (**D**–**F**) Statistical analysis of NLR performance at 5 min (**D**), 10 min (**E**), and the discrimination index (**F**). (**G**) Representative heatmaps of swimming paths during the MWM place navigation test on Day 5, where the small circles indicate the target platform location. (**H**) Escape latency curves during the 5-day training period. (**I**–**K**) Statistical analysis of the MWM probe trial, including platform crossing frequency (**I**), time spent in the target quadrant (**J**), and distance traveled in the target quadrant (**K**). Data are presented as mean ± SEM, *n* = 8. * *p* < 0.05, ** *p* < 0.01, *** *p* < 0.001 compared with the SCOP group.

To establish a causal link between the MsA-reshaped gut microbiota and cognitive improvement, we performed FMT from MsA-treated donor mice into SCOP-treated recipients (Figure 10A). In the NLR test, SCOP-treated mice exhibited significant spatial recognition deficits, characterized by reduced exploration of objects in novel positions. In contrast, MsA-FMT effectively reversed these impairments, as evidenced by significantly increased exploration performance and a higher discrimination index at both the 5- and 10-min intervals (Figure 10B–F). Representative trajectories highlighted a restored preference for novel locations in the MsA-FMT and DNP groups, suggesting that the cognitive protection associated with MsA can be largely recapitulated Via microbiota transplantation.

Further evaluation using the MWM supported the beneficial effects of MsA-FMT on spatial learning and memory. During the place navigation phase, SCOP-treated mice failed to develop efficient search strategies, resulting in disorganized swimming paths and significantly prolonged escape latencies. However, mice receiving MsA-FMT demonstrated a progressive optimization of their search patterns, with a significant reduction in escape latency over the 5-day training period, comparable to the DNP positive control (Figure 10G,H). In the probe trial, MsA-FMT mice displayed superior memory consolidation and retrieval, manifested by a significant increase in platform crossing frequency and a greater proportion of time and distance spent in the target quadrant (Figure 10I–K). These results collectively suggest that the MsA-modulated gut microbiota plays a vital regulatory role in mitigating SCOP-induced cognitive dysfunction.

## 4. Discussion

The clinical translation of cognitive-enhancing candidates is fundamentally determined by their biosafety and long-term tolerability. Currently, the application of first-line acetylcholinesterase inhibitors is often constrained by a narrow therapeutic window and significant adverse effects; for instance, tacrine has been largely withdrawn due to hepatotoxicity, while donepezil frequently induces peripheral cholinergic side effects such as nausea, emesis, and cardiac arrhythmias [29]. In contrast, MsA, a primary bioactive stilbene from *Morus alba* L., has emerged as a promising nutraceutical with an excellent safety record across diverse pathological models [30]. Previous studies have demonstrated that MsA, within a dose range of 5–50 mg/kg, exhibits no systemic toxicity and even exerts protective effects on the liver, kidneys, and heart by quenching oxidative stress and inhibiting apoptosis [21,31,32,33]. In the present study, we evaluated the therapeutic potential of MsA at doses of 10, 20, and 30 mg/kg/day. Our results confirmed that MsA was well-tolerated, with no significant alterations in body weight or organ indices. Notably, the 30 mg/kg/day dose (MsA-H) yielded the most robust and stable cognitive improvement in SCOP-treated mice, prompting its selection as the optimal dose for further mechanistic exploration.

The SCOP-induced amnesia model is a well-established paradigm for mimicking aspects of the early stages of AD, characterized by cholinergic dysfunction and memory impairment. Our behavioral assessments (NOR and MWM tests) demonstrated that MsA intervention effectively rehabilitated both non-spatial recognition memory and spatial reference memory. These findings align with our previous research showing that MsA can upregulate the expression of BDNF and CREB while alleviating neuronal damage in the hippocampus [22]. Importantly, the present study extends those findings by revealing that MsA not only preserves central neurotrophic signaling but also suppresses neuroinflammatory injury through a broader multi-system regulatory network. The restorative effects of MsA appear to be rooted in its dual capacity to neutralize oxidative stress and suppress neuroinflammation—two “accelerators” of AD-like progression [34]. By reducing MDA levels and bolstering the endogenous antioxidant defenses (SOD and GSH), MsA effectively mitigates lipid peroxidation in the cortex. Simultaneously, MsA suppressed microglial overactivation (Iba1+ cells) and downregulated pro-inflammatory cytokines (IL-1β, IL-6, and TNF-α), suggesting that its neuroprotective efficacy is mediated by the maintenance of neuroimmune homeostasis.

A growing body of evidence suggests that “leaky gut” and subsequent systemic inflammation play a pivotal role in the pathogenesis of neurodegenerative cognitive decline [12,35]. The intestinal barrier, primarily composed of tight junction proteins like ZO-1 and Occludin, serves as the first line of defense against the translocation of gut-derived neurotoxins [36,37]. In our study, SCOP-treated mice exhibited disruption of the intestinal architecture, accompanied by a marked loss of ZO-1 and Occludin. MsA intervention effectively reinforced these tight junctions and restored the villus-to-crypt (V/C) ratio. These findings indicate that MsA helps preserve intestinal barrier integrity and may thereby limit the entry of gut-derived inflammatory mediators into the systemic circulation. By maintaining intestinal homeostasis, MsA holds the potential to attenuate “bottom-up” inflammatory signaling to the brain, thereby presenting a plausible pathway to mitigate a key pathological route linking gut dysfunction to central neuroinflammation.

The core finding of this study is that MsA-mediated cognitive improvement is intrinsically linked to the remodeling of the gut microbial landscape. Our 16S rRNA sequencing revealed that MsA significantly reversed the SCOP-induced community shift, specifically by increasing the relative abundance of Bacteroidota and Actinobacteriota while reducing the expansion of Firmicutes. Although the Bacteroidota/Firmicutes (B/F) ratio is a broad ecological indicator, its restoration often reflects a transition from a pro-inflammatory “dysbiotic” state to a metabolic “homeostatic” state in AD models [38,39]. At the family and genus levels, MsA specifically enriched Muribaculaceae and *Dubosiella*. Muribaculaceae is recognized for its role in degrading complex polysaccharides and producing SCFAs, which are crucial for maintaining mucosal integrity and dampening systemic inflammation [40]. Emerging evidence highlights *Dubosiella* as a key beneficial genus associated with the regulation of oxidative stress and fatty acid metabolism in the brain [41,42]. Conversely, the suppression of opportunistic pathogens such as *Enterococcus* further suggests that MsA mitigates cognitive decline by reducing the “endogenous reservoir” of pro-inflammatory triggers within the gut.

To bridge the gap between gut dysbiosis and neuroprotection, we analyzed the hippocampal and cortical metabolomes. MsA significantly reprogrammed 64 core metabolites, primarily involved in choline metabolism, glutathione (GSH) synthesis, and phospholipid homeostasis. Choline is not only a precursor for acetylcholine but also a vital component of neural membranes; its restoration suggests that MsA supports both synaptic transmission and structural integrity [43]. In parallel, the observed changes in phospholipid and antioxidant-related metabolites indicate that MsA may counteract SCOP-induced membrane injury and redox imbalance at the metabolic level. Furthermore, the positive correlation between *Dubosiella* and neuroprotective lipids, such as PA (22:6/18:1), offers suggestive metabolic evidence of a statistical “crosstalk” between the gut and the brain, though further causal validation is required. Our data suggest that changes in the microbial ecosystem coincide with a reduced influx of systemic inflammatory signals alongside an increased abundance of essential lipids and antioxidants, which are associated with the shielding of neurons from SCOP-induced lipotoxicity and oxidative damage [44,45]. Consistently, our pathway enrichment analysis indicated that the identified biomarkers are associated with neurotransmission, redox balance, and lipid metabolism (Figure 8C). The enrichment of the cholinergic synapse pathway is consistent with the modulation of scopolamine-induced cholinergic blockade, supporting central neurotransmitter homeostasis. Concurrently, the representation of multiple lipid-related pathways, including fatty acid biosynthesis, fatty acid elongation, and fatty acid metabolism, points to alterations in neural membrane lipid dynamics. Moreover, the involvement of nicotinate and nicotinamide metabolism provides a metabolic link to the regulation of oxidative stress, given its role in maintaining cellular NAD+/NADH pools and mitochondrial redox defense. The integration of the mTOR signaling pathway and amino acid pathways (arginine, proline, and histidine metabolism) further indicates an impact on metabolic homeostasis [46,47]. Collectively, these metabolomic data indicate that MsA modulates multiple interconnected metabolic nodes, contributing to its neuroprotective effects.

We conducted FMT to explore the functional involvement of the intestinal microbiota in MsA-mediated neuroprotection. The observation that MsA-FMT successfully recapitulated the cognitive benefits—improving both spatial and recognition memory in recipient mice—suggests that the remodeled microbiota contributes significantly to the therapeutic outcomes, acting as a vital regulatory component of the observed efficacy. These findings align with the emerging paradigm that transplanting a “drug-optimized” microbiota can independently alleviate Alzheimer’s-like pathologies by bypassing direct pharmacological exposure [48,49]. Notably, in AD related models, FMT has been shown to bolster hippocampal synaptic plasticity and cholinergic tone by mitigating the systemic influx of gut-derived pro-inflammatory mediators [48,50]. Thus, our FMT results provide supportive evidence that gut microbial remodeling is intimately intertwined with, and strongly supports, the neuroprotective effects of MsA. By positioning the gut microbiota as a vital regulatory node for MsA’s cognitive-improving activity, our study underscores the transformative potential of microbiota-targeted strategies as viable therapeutic avenues for cognitive disorders.

Despite these promising findings, several limitations should be acknowledged. First, as a transient pharmacological screening model, the acute scopolamine-induced model replicates cholinergic and memory deficits but lacks the chronic, progressive pathology and organic, long-term accumulation of Aβ plaques and tau neurofibrillary tangles characteristic of transgenic AD models (e.g., APP/PS1). Because scopolamine directly suppresses gastrointestinal motility, the observed dysbiosis may partially reflect direct inhibition of gut transit. Crucially, this study exclusively utilized male ICR mice; given the established sex differences in both AD pathogenesis and gut microbiota composition, our findings warrant validation in female cohorts. Second, baseline constraints, including the absence of a SCOP-free MsA group and parallel vehicle/SCOP donor controls in the FMT experiment, limit our ability to completely decouple MsA-specific microbial effects from general microbiota reconstitution. Finally, the study lacks direct pharmacokinetic readouts, peripheral barrier evaluations (e.g., serum LPS), and tissue distribution data. Additionally, the sample size for intestinal histology and Western blot analyses (*n* = 3 per group) serves as an exploratory screening threshold; although intra-group variance was minimal, this limited replication may constrain statistical power and warrants scaling up in future confirmatory cohorts. Consequently, the precise gut-to-brain signaling pathways and the functional contributions of key taxa like *Dubosiella* require further elucidation through mono-colonization, metabolite supplementation, and nerve-blocking experiments.

## 5. Conclusions

In conclusion, MsA mitigates scopolamine-induced AD-like cognitive deficits by modulating the microbiota–gut–brain axis. MsA preserves intestinal barrier integrity, reshapes gut microbiota composition, particularly enriching *Dubosiella*, and regulates microbial metabolites, concomitantly mitigating neuroinflammation and oxidative stress in the brain. The FMT results further support a profound regulatory involvement of the reshaped gut microbiota in MsA-mediated neuroprotection. Collectively, these findings highlight MsA as a promising multi-target nutraceutical candidate for ameliorating AD-like cognitive impairment.

## Figures and Tables

**Table 1:** Nucleotide sequence of qPCR primer.

Target	Primer Sequence (5′ to 3′)	Primer Length (bp)
*IL-1β*	F: TCGCAGCAGCACATCAACAAGAG R: AGGTCCACGGGAAAGACACAGG	97
*IL-6*	F: CTTCTTGCGACTGATGCTGGTGAC R: AGGTCTGTTGGGAGTGGTATCCTC	94
*TNF-α*	F: GCCTCTTCTCATTCCTGCTTGTGG R: GTGGTTTGTGAGTGTGAGGGTCTG	149
*GAPDH*	F: AACTCCCACTCCTTCCACCTTCCG R: TCCACCACCCTGTTGCCTGTAG	113

Note: F (Forward), R (Reverse).

## Data Availability

The raw data supporting this article’s conclusions will be available upon request.

## Supplementary Materials

The following supporting information can be downloaded at: https://www.mdpi.com/article/10.3390/biology15131030/s1, Section S1.1 Raw membranes of Western blot with crop points; Section S1.2 Membranes employed in Western blot analysis; Section S2.1 Table S1. Main experimental reagents; Section S2.2 Table S2. Untargeted metabolomics demonstrate metabolite modulation by MsA in mice treated with SCOP in positive ion mode; Section S2.3 Table S3. Untargeted metabolomics demonstrate metabolite modulation by MsA in mice treated with SCOP in negative ion mode.

## Abbreviations

The following abbreviations are used in this manuscript:

AD	Alzheimer’s disease
MsA	Mulberroside A
BBB	Blood–brain barrier
MGBA	Microbiota-gut–brain axis
CNS	Central nervous system
SCFAs	Short-chain fatty acids
SCOP	Scopolamine
DNP	Donepezil
NOR	New object recognition
NLR	Novel location recognition
MWM	Morris water maze
FMT	Fecal microbiota transplantation
