# Supplementary material for: Mulberroside A Alleviates Scopolamine-Induced Cognitive Deficits by Suppressing Neuroinflammation and Oxidative Stress via the Dubosiella-Associated Microbiota–Gut–Brain Axis
Source: Biology (Basel). 2026 Jun 28;15(13):1030. doi: 10.3390/biology15131030 (PMC13359554; doi:10.3390/biology15131030)
Supplement: Supplementary file 1 [file biology-15-01030-s001.zip › biology-4363547-supplementary.pdf]

Supplementary Materials

Index

Content

	Page
**Section S1. The Effect of MsA on Scopolamine-Induced Mice**	2
**Section S1.1** Raw membranes of Western blot with crop points.	2
**Section S1.2** Membranes employed in Western blot analysis.	3
**Section S2. Supplementary Tables**	4
**Section S2.1** Table S1. Main experimental reagents.	4
**Section S2.2** Table S2. Untargeted metabolomics demonstrate metabolite modulation by MsA in mice treated with SCOP in positive ion mode.	5
**Section S2.3** Table S3. Untargeted metabolomics demonstrate metabolite modulation by MsA in mice treated with SCOP in negative ion mode.	6

Section S1. The Effect of MsA on Scopolamine-Induced Mice

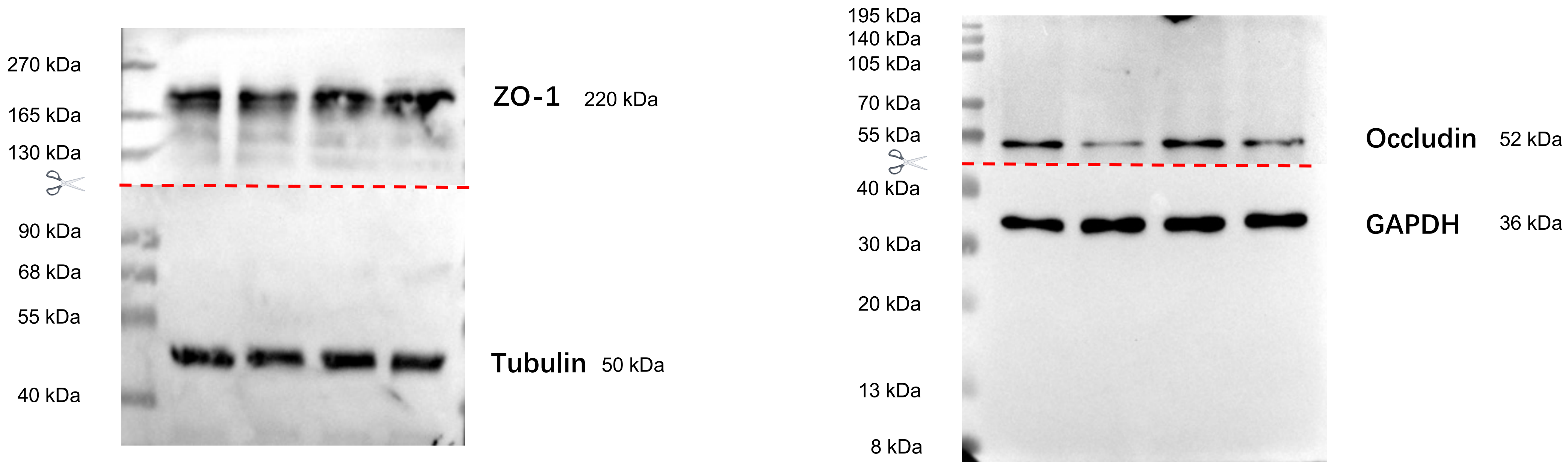

Figure 4F

Section S1.1 Raw membranes of Western blot with crop points

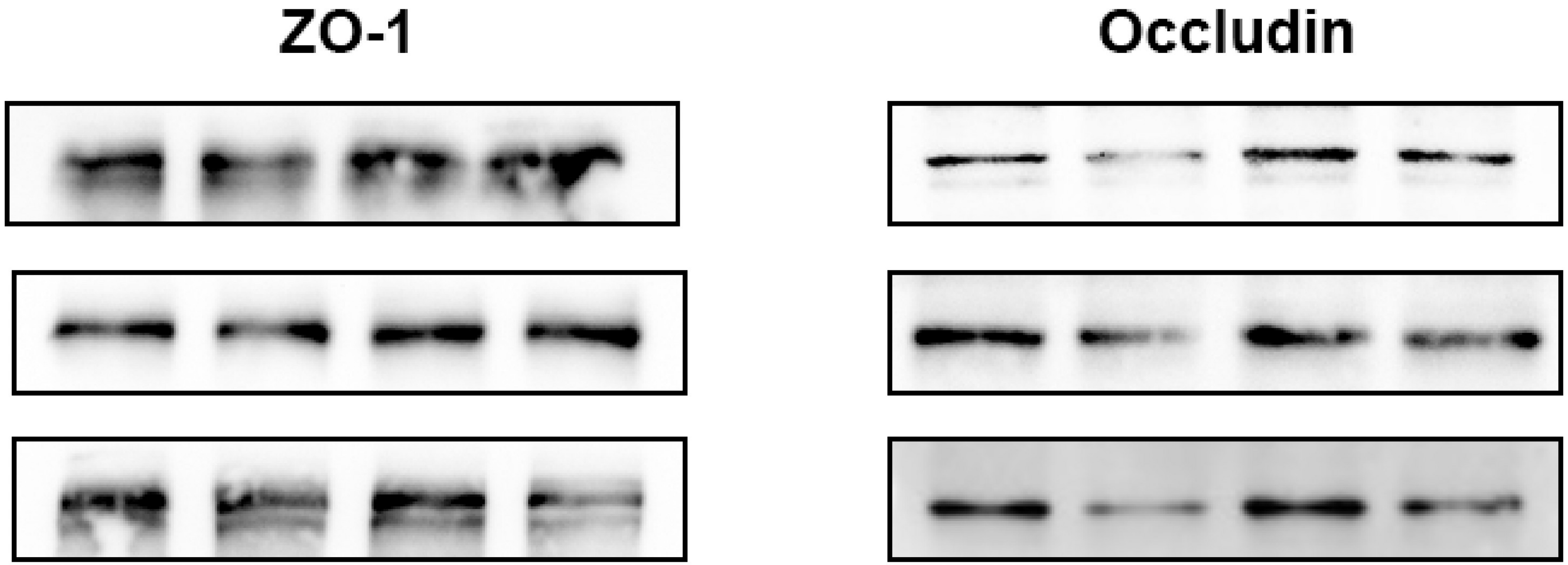

Figure 4F

Section S1.2 Replicates employed in the Western Blotting analysis

Section S2. Supplementary Tables

Section S2.1

Table S1. Main experimental reagents.

Reagent / Material	Catalog No.	Manufacturer
Malondialdehyde (MDA) assay kit	A003-1	Nanjing Jiancheng Bioengineering Institute (Nanjing, China)
Superoxide dismutase (SOD) assay kit	A001-3	Nanjing Jiancheng Bioengineering Institute (Nanjing, China)
Glutathione (GSH) assay kit	A006-2-1	Nanjing Jiancheng Bioengineering Institute (Nanjing, China)
TRIzol total RNA extraction reagent	R0016	Beyotime Biotechnology (Shanghai, China)
Reverse transcription kit	G3337	Servicebio Technology Co., Ltd. (Wuhan, China)
SYBR Green qPCR Master Mix	B690016	Sangon Biotech (Shanghai) Co., Ltd. (Shanghai, China)
RIPA lysis buffer	G2002	Servicebio Technology Co., Ltd. (Wuhan, China)
BCA protein assay kit	P0010	Beyotime Biotechnology (Shanghai, China)
Glycine	GC304019	Servicebio Technology Co., Ltd. (Wuhan, China)
Tris base	GC208001	Servicebio Technology Co., Ltd. (Wuhan, China)
Sodium dodecyl sulfate (SDS)	GC204005	Servicebio Technology Co., Ltd. (Wuhan, China)
5 × protein loading buffer	G2013	Servicebio Technology Co., Ltd. (Wuhan, China)
Phosphatase inhibitor cocktail	P1260	Beijing Solarbio Science & Technology Co., Ltd. (Beijing, China)
Phenylmethylsulfonyl fluoride (PMSF)	P0100	Beijing Solarbio Science & Technology Co., Ltd. (Beijing, China)
50 × TAE electrophoresis buffer	G3001	Servicebio Technology Co., Ltd. (Wuhan, China)
TBS buffer	G0001	Servicebio Technology Co., Ltd. (Wuhan, China)
50 × TAE electrophoresis buffer	G3001	Servicebio Technology Co., Ltd. (Wuhan, China)
Prestained protein marker	G2058	Servicebio Technology Co., Ltd. (Wuhan, China)
Prestained protein marker	G2089	Servicebio Technology Co., Ltd. (Wuhan, China)
Nitrocellulose membrane	HATF00010	Millipore (Billerica, MA, USA)
8% PAGE rapid preparation kit	G2042	Servicebio Technology Co., Ltd. (Wuhan, China)
12% PAGE rapid preparation kit	G2044	Servicebio Technology Co., Ltd. (Wuhan, China)
ECL detection reagent	AP34L024	Shanghai Liji Biotechnology Co., Ltd. (Shanghai, China)

Section S2.2

Table S2. Untargeted metabolomics demonstrate metabolite modulation by MsA in mice treated with SCOP in positive ion mode.

No.	Index	Level	Compounds	SCOP vs. Control			MsA vs. SCOP		
				VIP	FC	Trend	VIP	FC	Trend
1	ME0169549	1a	Niacinamide	1.88	0.85	↓**	2.03	1.17	↑*
2	MEDP0125	1a	Choline	1.87	0.82	↓**	1.92	1.21	↑*
3	ME0106189	1a	Creatinine	1.8	0.35	↓**	1.75	2.08	↑*
4	ME0117231	1b	1-Methylnicotinamide	2.27	2.18	↑**	2.04	0.55	↓**
5	ME0169676	1b	Pinolenic acid	1.62	2.65	↑*	1.62	0.43	↓*
6	MEDL00638	1b	Taurocholic acid	1.95	1.46	↑*	1.71	0.72	↓*
7	ME0109596	1b	Selenomethionine	1.66	1.19	↑*	2.28	0.68	↓**
8	MEDP0846	1b	4-Methylbenzoic acid	1.95	0.25	↓*	2.12	3.06	↑*
9	MEDTP01677	1b	FFA (12:1)	1.75	1.56	↑*	2	0.63	↓*
10	ME0108434	1b	3-Oxo-C8 (THF-3-yl) amide	1.62	1.53	↑*	1.73	0.72	↓*
11	MEDP1049	1b	2-Phenoxyethanol	2.16	1.88	↑**	2.01	0.62	↓**
12	ME0125626	1b	N-Acetyl-L-tryptophanamide	1.74	1.25	↑*	1.89	0.81	↓*
13	ME0125188	2	Mirtazapine	1.63	1.39	↑*	1.68	0.77	↓*
14	ME0126293	2	Pyrazinamide	2.04	0.82	↓**	1.92	1.15	↑*
15	ME0107371	3	Homocarnosine	2.11	2.01	↑*	1.77	0.56	↓*
16	ME0056964	3	PC (18:0/20:4)	2.2	3.16	↑**	1.96	1.71	↑*
17	ME0007803	3	Metoprolol	1.63	1.49	↑*	1.7	0.68	↓*
18	ME0150471	3	Gly-Pro-Ser	2.2	1.92	↑**	2.11	0.58	↓**
19	ME0054899	3	Methyl linoleate	1.7	1.65	↑*	1.69	0.58	↓*
20	ME0111434	3	β-Damascenone	1.71	1.38	↑*	1.75	0.78	↓*
21	ME0005721	3	5-Nonadecyl-1,3-benzenediol	2.22	5.08	↑*	1.84	0.29	↓*
22	ME0113264	3	5-Oxohexanal	2.1	1.94	↑**	2.16	0.53	↓**
23	ME0052959	3	Fusidic acid	1.8	1.19	↑*	2.41	0.69	↓**
24	ME0150781	1b	Palmitic acid	1.84	1.81	↑*	1.77	0.58	↓*
25	MEDP1662	2	N6,N6,N6-Trimethyl-L-lysine	1.58	0.81	↓*	1.65	1.22	↑*
26	MEDP1381	2	Carnitine C20:2	2.15	0.38	↓**	1.95	2.09	↑*
27	MEDP1422	2	Carnitine C8-OH	2	0.16	↓*	1.97	3.68	↑*
28	ME0165914	3	Monomethylsqualene	1.65	1.55	↑*	1.96	0.47	↓**
29	ME0194061	3	1-(Ammoniomethyl)cycloheaxaneacetate	2.09	3.11	↑**	1.98	0.47	↓**
30	ME0109311	3	Pro-Gln-Lys	1.79	0.64	↓*	2.13	1.83	↑**
31	ME0109073	3	Pepstatin A	1.68	1.75	↑*	2.02	0.5	↓**
32	MEDP1678	3	Thr-Val-Leu	1.67	2.05	↑*	1.57	0.62	↓*
33	ME0140438	3	P-HMF	1.96	1.79	↑*	2.16	0.49	↓**

Section S2.3

Table S3. Untargeted metabolomics demonstrate metabolite modulation by MsA in mice treated with SCOP in negative ion mode.

No.	Index	Level	Compounds	SCOP vs. Control			MsA vs. SCOP		
				VIP	FC	Trend	VIP	FC	Trend
1	ME0016406	1a	Carnosine	2.16	1.76	↑**	2.38	0.57	↓**
2	ME0056738	1a	FFA (16:1)	2.1	1.53	↑**	2.33	0.63	↓**
3	ME0013499	1a	2-Hydroxy-2-methylbutyric acid	2.29	3.5	↑**	1.95	0.43	↓*
4	ME0016123	1a	3-Hydroxyisovaleric acid	2.27	3.1	↑**	1.94	0.43	↓*
5	MEDN0416	1a	Ureidoisobutyric Acid	1.93	1.77	↑**	2.19	0.69	↓**
6	ME0053975	1a	Hydrocortisone acetate	1.6	2.35	↑*	1.56	0.37	↓**
7	ME0013504	1b	2-Hydroxy-3-Methylbutanoic Acid	2.24	3.56	↑**	1.84	0.45	↓*
8	ME0126754	1b	Theobromine	2.3	0.66	↓**	2.23	1.38	↑**
9	ME0105709	1b	Arginine	2.03	2.61	↑**	2	0.39	↓**
10	ME0117304	2	1-Pyrroline-4-hydroxy-2-carboxylate	1.67	1.24	↑*	1.6	0.76	↓*
11	ME0013531	2	2-Hydroxyhexadecanoic acid	2.24	0.37	↓**	2.57	3.56	↑**
12	ME0115233	2	Ribulose 5-phosphate	1.93	0.59	↓*	2.14	1.39	↑**
13	ME0103476	3	AICA ribonucleotide	2.1	2.54	↑*	1.72	0.55	↓*
14	ME0148594	3	Discadenine	1.63	1.88	↑*	1.67	0.56	↓*
15	ME0106506	3	DL-Glutamine	1.84	1.55	↑*	2.23	0.69	↓**
16	ME0143619	3	5-Formyl-THMPT	1.85	0.55	↓**	1.92	1.77	↑*
17	ME0105445	3	Agomelatine	1.83	0.39	↓*	2.02	3.36	↑*
18	ME0128358	3	(R)-2-benzylsuccinic acid	1.99	2.94	↑**	2.12	0.44	↓*
19	ME0195169	1a	GSH	1.55	0.77	↓*	1.8	1.37	↑*
20	ME0106328	1b	Cys-Phe	2.16	0.63	↓**	2.24	1.41	↑**
21	ME0012995	3	PC (18:0/22:6)	1.64	0.56	↓*	1.8	1.74	↑*
22	ME0055629	3	PA (16:0/22:5)	1.81	0.48	↓*	2.08	3.11	↑**
23	ME0056361	3	PA (22:6/18:1)	1.88	0.28	↓**	1.93	4.02	↑*
24	ME0133192	3	3-O-alpha-mycarosylerythronolide B	1.77	2.49	↑**	2.38	0.5	↓**
25	ME0059297	3	PE-NMe (15:0/18:0)	2.44	11.45	↑**	2.41	0.16	↓**
26	ME0014354	3	C22:5-ynoic acid	1.6	1.39	↑*	2.21	0.65	↓*
27	ME0012966	3	PA (16:0/18:2)	1.86	0.31	↓*	1.65	2.71	↑*
28	ME0054509	3	LPA (8:0)	1.67	0.56	↓*	2.5	1.93	↑**
29	ME0141121	3	1,2-Dinitrobenzene	1.66	2.06	↑*	1.67	0.56	↓*
30	ME0154464	3	N-Glycolyl-D-mannosamine 6-phosphate	2.15	2.35	↑**	2.02	0.48	↓*
31	ME0143328	3	GSH-HNE	1.67	0.38	↓*	1.73	2.2	↑*

Note: Metabolite identification levels were assigned according to the Metabolomics Standards Initiative guidelines: Level 1a, identified by matching accurate mass (*m*/*z*), MS/MS fragmentation patterns, and retention time with in-house authentic standards; Level 1b, putatively identified by matching MS/MS spectra with standard libraries; Level 2, putatively annotated compounds based on MS/MS spectral similarity search against public databases; Level 3, putatively characterized compound classes.
